# Large, prolonged flooding and pool persistence promote floodplain fish diversity in a threatened river

**DOI:** 10.1002/eap.70155

**Published:** 2025-11-25

**Authors:** Oliver P. Pratt, Leah S. Beesley, Daniel C. Gwinn, Thiaggo C. Tayer, Bradley J. Pusey, Chris S. Keogh, Samantha A. Setterfield, Michael M. Douglas

**Affiliations:** ^1^ School of Agriculture and Environment The University of Western Australia Perth Australia; ^2^ Biometric Research South Fremantle Australia

**Keywords:** alpha diversity, Bayesian, floodplain, intermittent, lateral connectivity, occupancy model, river‐floodplain connectivity, riverine, tropical, water

## Abstract

While it is widely recognized that reduced river‐floodplain connectivity has contributed to the decline of biodiversity in floodplain rivers, surprisingly few studies have quantified the relationship between connectivity, pool persistence, and fish assemblage structure to the level required to generate measurable targets for management. The task is further complicated by the inherent complexity of accurately describing fish assemblages. We maximized our capacity to describe unbiased hydrology–fish relationships by sampling fish assemblages in floodplain pools with a variety of connection histories (60 sampling events), and by using a hierarchical multispecies occupancy model that accounts for changes in sampling design and species detection. Our study was conducted in a tropical wet‐dry river threatened by water resource development and elevated temperatures associated with climate change, the Fitzroy River (Western Australia). Our results revealed that wet season (river‐floodplain connectivity) and dry season (pool persistence) components of the hydrological cycle influenced fish occurrence in floodplain pools. Pools that were connected to the river by short distances were substantially more species rich than distal pools. This effect was strong at distances <2000 m but negligible at distances greater than 3000 m. Species richness in floodplain pools increased when wet season connection to the river lasted more than 25 days, and when river stage height exceeded 6 m. Prolonged connection to the river (up to 90 days) during overbank flooding (river stage height >11 m) maximized fish species richness in floodplain pools. Dry season components of the hydrological cycle also influenced fish assemblage structure, with pools that persisted during the preceding dry season twice as species rich as those that dried. Our model revealed that sampling gear influenced species detectability, indicating that accounting for variable detection is critical when assessing fish assemblage structure. Given that large flood events are less likely to be impacted by water take, we recommend that managers seeking to maintain floodplain fish diversity ensure that water resource development does not negatively impact pool persistence during the dry season.

## INTRODUCTION

Freshwater biodiversity has decreased dramatically over the last 50 years (Albert et al., 2021; Harrison et al., 2018) and water resource development and climate change are leading contributors to this decline (Dudgeon, 2019; Lintermans et al., 2024; Reid et al., 2019; Scherer et al., 2023). Water extraction and factors associated with a shifting climate, such as increased drying or changing rainfall patterns, alter natural river flow and disrupt seasonal cycles of flooding and drying (Döll & Schmied, 2012; Vogl & Lopes, 2009). These changes to the natural flow regime, that is, the magnitude, timing, frequency, and duration of flow, can impact the physical habitat of a river and the ecological processes that support biodiversity (Bunn & Arthington, 2002; Poff et al., 1997).

Hydrological connectivity is one ecological process commonly impacted by alteration of the natural flow regime. In lowland rivers with well‐developed floodplains, flow alteration typically leads to a marked reduction in lateral connectivity, that is, between the river and its floodplain (Bowen et al., 2003). Reduced lateral connectivity can restrict access to the floodplain for fish and other aquatic biota (Amoros & Bornette, 2002) causing a decline in species richness in floodplain pools (Bolland et al., 2012; Jiang et al., 2021). Decreased lateral connectivity can also impact pool persistence because many floodplain waterbodies will dry if not periodically refilled during lateral connectivity events that is, flooding. Pool drying can lead to deteriorating water quality, habitat loss, and increased competition for resources which can influence species assemblage structure (Arthington et al., 2005; Magoulick & Kobza, 2003; Sargent & Galat, 2002), while complete drying results in extirpation of many aquatic biota. The effect of drying on species assemblages is likely to be further exacerbated in regions where temperatures are expected to increase due to climate change. Floodplain waterbodies contribute substantially to riverine biodiversity and functioning (Jardine et al., 2015; Junk et al., 1989; Sullivan & Watzin, 2009; Welcomme, 2000), thus it is important to maintain effective lateral connectivity to facilitate access to and from the floodplain for aquatic biota such as fish and ensure natural cycles of pool drying and filling are not unduly impacted by water resource development and climate change.

To protect and minimize damage to the ecological integrity of river systems undergoing water extraction, it is imperative to set and work towards meaningful ecological targets (Richter et al., 2003). While considerable literature explores the relationship between lateral connectivity, pool persistence, and floodplain fish assemblage structure, few studies quantitatively define these relationships to the level required to generate measurable targets for management. For instance, many studies categorize connectivity categorically (e.g., permanently connected, intermittently connected, or disconnected) (Liu & Wang, 2018; Manfrin et al., 2020) or use ordinal or semi‐quantitative scales (Bolland et al., 2012; Miranda, 2005). Furthermore, the statistical approaches employed in many studies preclude the ability to simultaneously predict fish assemblages and single species occurrence across a gradient of lateral connectivity (Lear et al., 2023; Penha et al., 2017). This means that predictions regarding the likely outcomes of different water take scenarios or future climate projections cannot be readily assessed (Cuddington et al., 2013).

The relationship between lateral connectivity, pool persistence, and fish assemblage structure in floodplain waterbodies is complex. Lateral connectivity interacts with species dispersal capacities to directly influence the number and type of potential colonists (Heino et al., 2015). Pools far from the river main stem are unlikely to be colonized by species with poor dispersal capacities, though permanent floodplain waterbodies may act as important “stepping stone” habitats (Holt, 1993; Magoulick & Kobza, 2003). Historical conditions such as antecedent flow patterns can affect fish assemblage structure by shaping the spatial distribution of species across the floodplain and influencing recruitment dynamics (Fornaroli et al., 2020; Humphries et al., 1999; Kennard et al., 2007). Niche habitat preferences, species adaptations, and life‐history strategy can also play an important role (Chen et al., 2022; Hoeinghaus et al., 2003; Winemiller, 1989). Disentangling these relationships is vital for understanding the key processes shaping fish assemblages in floodplain pools.

The main drivers of fish assemblage structure can be further obscured by sampling methods, experimental design, and chosen analytical approach. For instance, detection of fish is imperfect and can be influenced by a variety of factors including sampling gear (see Gwinn, Beesley, et al. (2016) for a review). Many researchers seeking to describe the entire fish assemblage employ multiple gear types and standardize effort (e.g., by area sampled, time period, or post‐sampling techniques such as catch‐per‐unit‐effort) in an attempt to overcome this issue (Haynes et al., 2013). However, confidence in these solutions can be misplaced because the efficacy of gears will vary with species (Beesley et al., 2014; Wedderburn, 2018), water depth, and water quality (Lyon et al., 2014; Pierce et al., 1990). If not mathematically accounted for, variable detection can lead to biased or spurious estimates of the fish assemblage (Gwinn, Allen, et al., 2016; Gwinn, Beesley, et al., 2016; White et al., 2020). Unfortunately, many statistical approaches common in the freshwater literature do not account for changes in sampling design or imperfect detection. Therefore, changes in species occurrence are confounded with changes in sampling design and efficiency, which can lead to type 1 errors (Archaux et al., 2012). Moreover, these statistical techniques often provide inaccurate estimates of species occurrence where there are limited data, as is often the case with rare or rarely detected species. Indeed, it is common to see data‐limited species excluded from analysis altogether, despite rare species often being significant to management, for example, threatened species (Lear et al., 2023; Poos & Jackson, 2012).

Hierarchical multispecies occupancy models (HMSOM) are a means to analyze species‐specific and community‐level ecological data while accounting for imperfect detection (Dorazio & Royle, 2005; Dorazio et al., 2006). The occurrence of species is modeled explicitly and separately from the sampling process meaning that rare species can be distinguished from rarely detected species (Gwinn, Allen, et al., 2016). Hierarchical models are advantageous where data are sparse (i.e., rare or rarely detected species) because species‐level estimates are structured as random effects and “borrow strength” from community‐level means (Gelman & Hill, 2006; Zipkin et al., 2010). Community‐level hyper‐parameters that describe variation among species (i.e., covariates) typically have high precision because the model aggregates data from among species (Sauer & Link, 2002). The method also allows for changes in sampling design so that sites requiring greater sampling effort or different sampling methods to adequately describe fish assemblage structure can be easily accommodated. This flexibility means hierarchical occupancy models are an ideal choice for variable management scenarios.

In this study we use data collected from 4 years of fish surveys in floodplain pools of a river threatened by water resource development and climate change, the Fitzroy River in Western Australia, to model the relationship between fish occurrence and a suite of lateral connectivity metrics, pool persistence, and antecedent flow. We use a hierarchical multispecies occupancy modeling approach that accounts for changes in sampling design and species‐specific differences in detection, deriving unbiased estimates of fish assemblage structure. Our aim was to identify the main drivers of fish assemblage structure in floodplain pools and to quantify these relationships in order to provide managers with better information about the likely consequences of alterations to the natural flow regime.

## METHODS

### Study area

**FIGURE 1:**
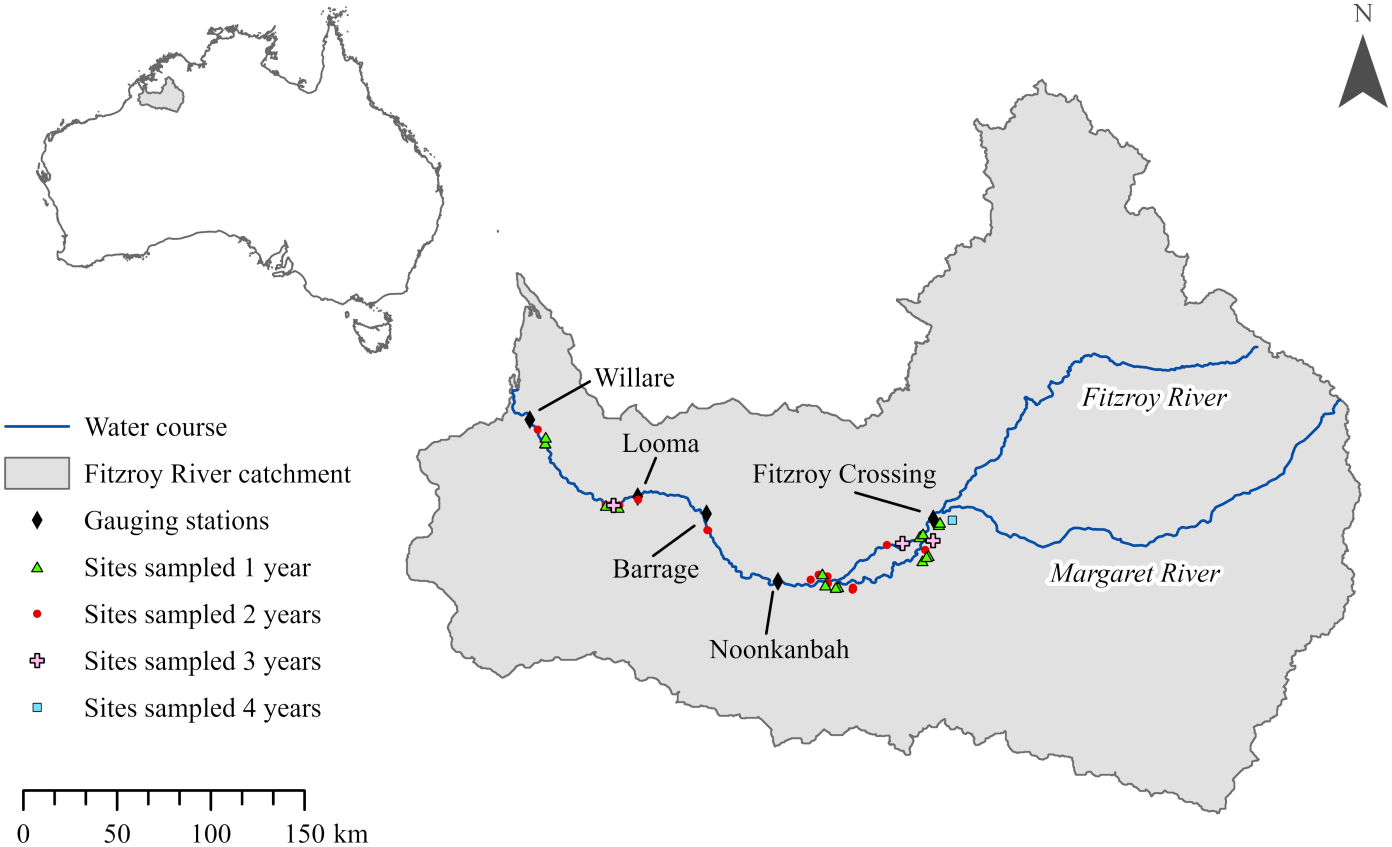
Location and frequency of sites (floodplain pools) sampled and gauging stations (diamonds, labeled) in the Fitzroy River, Kimberley, Western Australia. Triangles represent sites sampled in 1 year (*n* = 17), circles in 2 years (*n* = 15), crosses in 3 years (*n* = 3), and squares in all 4 years (*n* = 1). Inset shows location of the Fitzroy River catchment in Australia.

The Fitzroy River is located within a savannah landscape in the wet‐dry tropical region of the Kimberley, Western Australia (Figure 1). The river (700 km in length, catchment area 94,000 km^2^) has an annual mean discharge of 6600 GL (Petheram et al., 2018). The lowland section of the river is characterized by a sinuous channel with occasional large anabranches (Taylor, 1999). The flow regime is classified as wet season highly intermittent (class 10) by Kennard et al. (2010). Wet and dry seasons are distinct and predictable in incidence but not quantity which can lead to extreme (both high and low) periods of water availability across the catchment. Climate modeling indicates the region is expected to experience prolonged periods of high temperatures during the dry season (Suppiah et al., 2007), which will likely increase evaporation rates and reduce the persistence of floodplain pools (Hobday & Lough, 2011). During the dry season (~May–November), the main channel disaggregates into a series of deep pools several kilometers long connected by shallow runs. The floodplain is characterized by a patchwork of spatially distinct pools formed in distributary creeks and depressions that contract under drying conditions with many becoming increasingly turbid over time. Many of these pools are semi‐permanent and will dry completely resulting in the extirpation of aquatic organisms, while others will persist until hydrological connectivity is restored in the following wet season. Wet season flows (~December–April) connect main channel pools and backfill floodplain distributary creeks, facilitating the passage of aquatic biota. Overbank flows which inundate the floodplain are relatively brief (days–weeks) compared with other large rivers in the wet‐dry tropics where water may remain on the floodplain for several months (Jardine, Pettit, et al., 2012). Flow in the river is largely unmodified except for one main regulatory structure, a 3 m‐high barrage, located midway along the river (Figure 1). However, the water resources of the Fitzroy River have been earmarked for the development of irrigated agriculture (Petheram et al., 2018) which has the potential to reduce lateral connectivity, lessening the area, depth, and duration of seasonal floodplain inundation (Karim et al., 2018). Permanent and semi‐permanent floodplain pools provide an important contribution to landscape scale biodiversity and main channel ecosystem functioning (Beesley et al., 2022; Lear et al., 2023; Pratt et al., 2023, 2024), and there is evidence that hydrological metrics associated with flood magnitude may influence species diversity and species‐specific recruitment in one permanent deep‐water floodplain creek (Lear et al., 2023). However, currently there is limited knowledge regarding the factors that structure fish communities more broadly across the floodplain. It is likely that both wet season (lateral connectivity) and dry season (pool persistence) components of the hydrological cycle will influence fish assemblages. This information has the potential to guide water allocation planning in the Fitzroy River to protect important flows, floodplain biota, and ecological processes from future water resource development and climate change.

### Study design and sampling methods

Fish were sampled in floodplain pools during dry conditions between June and December, over 4 years (2018–2021). A total of 36 sites were sampled spanning the lower 350 km of the river between Fitzroy Crossing and Willare (Figure 1). A subset of sites was sampled over multiple years giving a total of 60 site‐sampling events. Floodplain pools ranged from highly ephemeral to semi‐permanent and remained hydrologically disconnected from one another and the main channel during the dry season. The fish assemblage at each site was quantitatively sampled using one or more active methods including boat electrofishing and three seine net configurations. The choice of sampling method was based on pool morphology (e.g., water depth, pool size) and saltwater crocodile risk. To maximize capture probability across fish species, all available habitats were sampled (substrate, structural complexity, depth). Sampling effort was dictated by pool size and habitat complexity, such that large pools with multiple within‐site habitats received greater sampling effort. Boat electrofishing used a 4.4 × 1.8 m aluminium hull electrofishing boat fitted with a Mid‐West Lakes HC80 electrofishing unit. The unit was typically operated at 300 volts DC (range 180–750 volts), 120 pulses per second, 25% duty cycle and 25 A (range 10–30 A). Settings were adjusted at each site relative to water conductivity. One operator collected fish from the bow of the boat with a dip net (9 mm mesh size). Sampling effort was defined as the time (in seconds) that current was applied to the water. A 10 m‐long seine net was used perpendicular to the bank in shallow pools or the shallow margins of deeper pools. A 5 m‐long net was used in small pools or where complex habitat restricted the use of the large net. Both nets had a 1 m drop and 9 mm mesh. A 7 m‐long net with a 1 m drop and 2 mm mesh was used to target smaller‐bodied fish. Sampling effort was defined as the distance (m) of net drag for all seine net configurations. All fish collected were identified to species level and released at the approximate point of capture.

Environmental parameters known to influence species detection were measured at each site prior to fish sampling. Mean turbidity (NTU) and electrical conductivity (in microsiemens per liter) were estimated from measurements taken at three locations within each site using a YSI ProDSS (Xylem, U.S.A) held at a depth of 15 cm. Pool maximum depth (in meters) was estimated from a series of point measurements along transects (*n* = 2–5 per site). Measurements were spaced 2–5 m apart in accordance with pool size and depth heterogeneity.

Remote sensing methods were used to collect data describing wet season (lateral connectivity) and dry season (pool persistence) processes known to influence fish assemblage structure in floodplain waterbodies. These included: whether the floodplain pool connected to permanent water (e.g., the river or permanent floodplain pool) during the preceding wet season (0,1), the minimum distance between the floodplain pool and permanent water along flow pathways during peak flood (in meters) (Meitzen et al., 2018), the duration of hydrological connection between floodplain pools and permanent water (in days) (Janáč et al., 2010), maximum main channel stage height (in meters) (Lear et al., 2023), whether pools persisted or dried prior to connection (0,1) (Beesley & Prince, 2010), and antecedent flow conditions measured as total main channel discharge (GL) recorded two wet seasons prior (Kennard et al., 2007). For instance, for a pool that was sampled in the dry season after the 2020/2021 wet season, two wet seasons prior refers to total main channel discharge during the 2019/2020 wet season. This metric of antecedent flow condition was used because many of the target species are short‐lived, mature quickly, and show variable recruitment linked to spawning guild influenced by total wet season discharge (Pusey et al., 2017; Lear et al., 2023). Consequently, shifts in assemblage structure driven by recruitment pulses are expected to be most pronounced within 1–2 years. Visual analysis of daily satellite images (Planet Team, 2017) was conducted to determine whether pools connected to permanent water, the duration of connectivity between floodplain pools and permanent water, and whether pools persisted throughout the previous dry season. Maximum main channel stage height data and total discharge data two wet seasons prior were collected from the closest gauging station to each site (Figure 1). All gauging station data were provided by the Australian Bureau of Meteorology. The minimum distance between floodplain pools and permanent water was estimated using cost distance analysis to derive the “least cost path.” This was a three‐step process where first satellite images (4‐band RGB and NIR: 3 m resolution) coinciding with peak flood in the area of each site were acquired from Planet Team (2017). The timing of peak flood was determined from maximum river stage height data derived from the closest gauging station to each site. We then generated binary water maps from satellite images where 0 = no water and 1 = water, as per Tayer et al. (2023). Tayer et al. (2023) demonstrated this approach is both accurate and precise for mapping water on inundated floodplain in the Fitzroy River. Each water map was manually inspected to ensure hydrological connection pathways were not erroneously disconnected by objects such as overhanging vegetation or cloud cover. Finally, we used cost distance, back‐link and cost path to polyline tools within software ArcPro version 2.7 to map the shortest distance or “least cost path” between permanent water and floodplain pools along hydrological connection pathways. The river main stem was the closest permanent water source to each site in all instances but for one pool; therefore hereafter we use the term “river” to refer to permanent water.

### Model structure

We used a hierarchical multispecies occupancy model (Dorazio & Royle, 2005; Dorazio et al., 2006) to investigate the relationships between wet season (lateral connectivity) and dry season (pool persistence) components of the hydrological cycle, and fish species occurrence in floodplain pools. This method allowed for simultaneous estimation of species‐specific and community‐level covariate effects (e.g., lateral connectivity metrics) while accounting for imperfect detection across species and gear type. Detection probability was modeled explicitly and separately from occurrence (i.e., the probability a species was present at a site), deriving unbiased estimates of species occupancy.

Our model describes site‐level occurrence of fish species as:

$$z\left(i,j\right)\sim \text{Bernoulli}\left(\psi_{i,j}\right),$$

where the occurrence of species i at site j is an imperfectly observed (latent) random variable, $z\left(i,j\right)$, which is the outcome of a Bernoulli trial where $\psi_{i,j}$ is the probability that species i occurs at site j with $z\left(i,j\right)=1$ if it does occur and zero if not. True occurrence is imperfectly observed (Gwinn, Beesley, et al., 2016); by using replicated survey data the true absence of a species from any given site can be distinguished from its nondetection, specified through a detection model (MacKenzie et al., 2002). This assumes the system is closed to changes in occupancy (e.g., colonization) during the period of replicate sampling. We defined the detection model for species i at site j during replicate k as:

$$y\left(i,j,k\right)\sim \text{Bernoulli}\left(p_{i,j,k}\times z\left(i,j\right)\right),$$

where $y\left(i,j,k\right)$ represents incidence detection data with 1 indicating that species i was detected at site j in replicate k, $p_{i,j,k}$ is the detection probability of species i for the *k*th replicate at site j, given species i is present at site j. Inclusion of the latent occurrence state in the specified detection model satisfies the condition that detection is zero when a species is not present at a particular site because $z\left(i,j\right)=0$ (MacKenzie et al., 2002; Zipkin et al., 2010).

We expected that the occupancy status of species i at site j would vary as a function of components of the hydrological cycle. Thus, we incorporated covariates into the occurrence model to investigate the influence of measured lateral connectivity metrics and hydrological metrics using a logit link specified as:

$$\text{logit}\left(\psi_{i,j}\right)=\beta_{1,i}+\beta_{2,i}C_{j}+\beta_{3,i}C_{j}F_{j}+\beta_{4,i}D_{j}+\beta_{5,i}S_{j}+\beta_{6,i}P_{j}+\beta_{7,i}P_{j}A_{j}+\varepsilon_{i,j}^{\text{site}}+\varepsilon_{i,j}^{\text{year}}+\varepsilon_{i,j}^{\text{gauge}},$$

where $\beta_{1,i}$ is the species‐specific intercept and $\beta_{2,i}–\beta_{7,i}$ are species‐specific covariate effects on occurrence. Variables C,D, S, and P represent pool connection to the river during the preceding wet season (0,1), duration of connectivity, maximum river stage height, and pool persistence (0,1), respectively. The term $\beta_{3,i}C_{j}F_{j}$ models the influence of distance between floodplain pools and the river along flow pathways $F_{j}$, which is only relevant if connection is established between the two habitats during flood events ($C_{j}$). The term $\beta_{7,i}P_{j}A_{j}$ models the influence of total main channel discharge two wet seasons prior ($A_{j}$), which is only pertinent if the pool persisted ($P_{j}$) throughout the following dry season. Correlation between covariates was assessed using Pearson correlation coefficients which were all <0.7. Prior to analysis, continuous covariates were natural log transformed and standardized, that is, centered on zero and scaled to 1 standard deviation (SD). The parameters $\varepsilon_{i,j}^{\text{site}}$ and $\varepsilon_{i,j}^{\text{year}}$ are species‐specific random effects across sites to account for non‐independence for sites sampled on multiple years and the expectation of non‐independence between years, respectively. We also expected that sites clustered around each of the five gauging stations would be more similar than between gauging stations due to landscape morphology (e.g., slope) and channel bathymetry (Karim et al., 2016). To account for this non‐independence, we included the parameter $\varepsilon_{i,j}^{\text{gauge}}$ as a species‐specific random effect across sites.

We expected that detection probabilities would vary across species with gear type and that the efficiency of the sampling gear would be influenced by sampling effort and environmental factors. To account for this, we incorporated covariates into the detection model with a logit link as:

$$\text{logit}\left(p_{i,j,k}\right)=\beta_{1,i}+\beta_{2,i}E_{k}+\beta_{3,i}W_{k}+\beta_{4,i}W_{k}^{2}+\beta_{5,i}T_{k}+\beta_{6,i}T_{k}^{2}+\beta_{7,i}N_{k}+\beta_{8,i}C_{k}$$

where $\beta_{1,i}$ is the species‐specific intercept and $\beta_{2,i}–\beta_{8,i}$ are gear‐specific covariate effects of variables that are known to influence detection. This included effort $E_{k}$ (Pritt et al., 2014), maximum water depth of each sampling event $W_{k}$ (Haynes et al., 2013), and turbidity $T_{k}$ (Lyon et al., 2014). In addition to main effects, covariates W and T were also formulated as quadratic terms (e.g., $W_{k}^{2}$) because the relationships are likely to be asymptotic (Gwinn et al., 2024; Korman & Yard, 2017). We incorporated two additional covariates for boat e‐fishing, $N_{k}$ and $C_{k}$, to account for the influence of different netting personnel and electrical conductivity, respectively, on sampling efficiency (Reynolds & Dean, 2020). Correlation between covariates in the detection model was assessed using Pearson correlation coefficients which were all <0.7. All continuous covariates were centered on zero and scaled to 1 SD prior to analysis. Effort $E_{k}$, was natural log transformed prior to standardization.

The hierarchical component of the model linked both occurrence and detection processes across species by treating species‐specific and gear‐specific covariate parameters as random effects governed by community‐level hyper‐parameters. For example, in the occurrence model we assumed that species‐level parameters were drawn from a normal distribution described by a community mean (μ) and community SD (σ) for example, $\beta_{2-7,i}\sim \text{Normal}\left(\mu_{2-7}^{\beta },\sigma_{2-7}^{\beta }\right)$. A major benefit of the multispecies hierarchical approach is that data are shared across species, improving the precision of both species‐specific and community level estimates (Gelman & Hill, 2006).

### Model selection

Effective management of any ecosystem requires an understanding of both community‐level and species‐specific responses to environmental stressors. We expected that different species would show variable responses to our connectivity and persistence metrics. We also considered it likely that certain metrics would be more influential across all species than others. To provide information regarding the community response to our various metrics, we conducted model selection at the community level using a Bayesian mixture modeling approach known as stochastic search variable selection (SSVS) (Burton et al., 2012; George & Mcculloch, 1993; O'Hara & Sillanpää, 2009). This method invokes parameter shrinkage using a hierarchical structure where each covariate is multiplied by a latent random‐effect inclusion parameter (w) which takes the value or either 0 or 1. We specified a Bernoulli prior for each inclusion parameter of 0.5 that assigns equal prior probability of inclusion or exclusion of each covariate in the model. The posterior mean of each inclusion parameter can be interpreted as support for a non‐zero parameter value. The predictive properties of the model are optimized under SSVS because effect estimates are automatically model averaged (George & Mcculloch, 1993; O'Hara & Sillanpää, 2009). We also assessed the importance of covariates on individual species occurrence by inspecting the posterior distributions of all parameters. We considered covariate effects statistically different from zero when 90% Bayesian credible intervals of posterior samples did not include zero (approximating α≤0.1). In addition to Bayesian credible intervals, we also used posterior probabilities as evidence for the influence of model covariates. For instance, the proportion of the posterior sample that is greater than zero is directly related to the probability that the covariate has an effect greater than zero. This technique facilitates exploration of covariate effects beyond the binary “significant at α≤0.05or0.1” common to frequentist methods and thus provides a more nuanced approach to interpreting model results.

### Model fitting methods

The occupancy model was fitted within a Bayesian framework where posterior probability distributions were estimated using the Gibbs sampler JAGS version 4.3.0 (Plummer, 2003) called with program R version 4.1.2 (R Core Team, 2015) with package R2jags version 0.7‐1 (Su & Yajima, 2021). We opted for a Bayesian approach because it allows full propagation of uncertainty in parameter estimates, offers flexible tools for model selection and regularization, and facilitates the implementation of complex hierarchical models, compared to frequentist methods (Kéry, 2010; O'Hara & Sillanpää, 2009). Two MCMC chains were run for 100,000 iterations with a thin rate of 10. The first 50,000 iterations were discarded leaving a posterior sample of 10,000 for inference. MCMC chains were considered converged when the $\hat{R}<1.1$ (Gelman et al., 2004). All logit scale parameters (covariate parameters in occurrence and detection models) were given non‐informative student *t*‐distributions with σ=1.566 and ν=7.763 as per Dorazio et al. (2011). Priors on all variance hyper‐parameters were given weakly informative half student *t*‐distributions with σ=1.8 and ν=2 as per Beesley et al. (2022). A weakly informative prior provides minimal information based on the realities of the data and model while having negligible influence on the shape of the posterior distributions (Gelman et al., 2004; Soto‐Shoender et al., 2020). The benefit of weakly informative priors is two‐fold. Firstly, they improve model stability and aid convergence of MCMC chains. Secondly, they lead to parameter estimates which are closer to zero than maximum likelihood estimates, thus providing a form of model regularization which improves predictive performance (Broms et al., 2016). We conducted a posterior‐predictive check using a Bayesian *p*‐value to evaluate model fit (Kéry, 2010; Soto‐Shoender et al., 2020). This method provides a measure of over or under dispersion of the data relative to the model. Pearson residuals were calculated for the observed data as well as data which were simulated directly from the model and were thus considered “perfect” because all model assumptions were met (Kéry, 2010). We derived a fit statistic that is equal to 1 when the Pearson residual was greater for the observed data than the simulated data and zero otherwise. The mean value of the posterior of the fit statistic is the Bayesian *p*‐value. A value of 0.5 indicates perfect model fit and values between 0.05–0.95 indicate adequate fit (Soto‐Shoender et al., 2020).

## RESULTS

**FIGURE 2:**
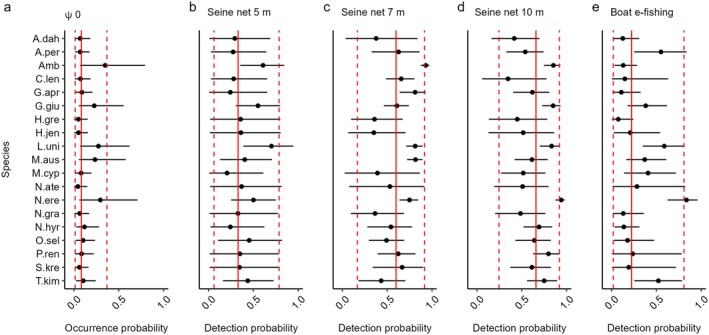
Community and species‐specific model estimates of (a) occurrence probability, (b) detection probability of 5 m seine net, (c) detection probability of 7 m seine net (d) detection probability of 10 m seine net, and (e) detection probability of boat e‐fishing. The solid line represents the mean effect at the community level and dashed lines the 90% SE. Error bars indicate 90% Bayesian credible intervals at the species level. See Table 1 for species codes.

A total of 35,808 individuals from 19 species was detected (a “naïve” measure of occurrence without accounting for detectability; MacKenzie et al., 2002) across the 60 sampling events (Table 1). Most individuals detected belonged to four taxa (*Ambassis* spp., *Leiopotherapon unicolor*, *Melanotaenia australis*, and *Nematalosa erebi*) which represented 94% of total catch. These species were also the most widespread and were detected in at least 62% of sampling events (Table 1). One species, *Neosilurus ater*, was detected at only one site where five individuals were caught. All other species were detected in a minimum of three sampling events. Model results indicate mean occurrence probabilities ranged from 0.348 for *Ambassis* spp. to between 0.044 and 0.061 for less common species such as *N. ater*, *Hephaestus jenkinsi, Hannia greenwayi*, and *Strongylura krefftii* (Figure 2a). Convergence of model parameters was satisfactory ($\hat{R}<1.1$) and Bayesian *p*‐values, which ranged from 0.420 to 0.529, indicated adequate model fit for the detection sub‐model (see Appendix S1: Figure S1).

**TABLE 1:** Species detected in floodplain pools of the Fitzroy River between 2018 and 2021 across 60 site‐sampling events.

Code	Species name	Common name	Site‐sampling detections	Individuals caught
A.dah	*Anodontiglanis dahli*	Toothless catfish	6	14
A.per	*Amniataba percoides*	Barred grunter	14	269
Amb	*Ambassis* spp.	Northwest glassfish	56	23,420
C.len	*Craterocephalus lentiginosus*	Prince Regent hardyhead	11	234
G.apr	*Glossamia aprion*	Mouth almighty	16	311
G.giu	*Glossogobius giuris*	Flathead goby	39	485
H.gre	*Hannia greenwayi*	Greenway's grunter	4	7
H.jen	*Hephaestus jenkinsi*	Western sooty grunter	3	5
L.uni	*Leiopotherapon unicolor*	Spangled perch	42	1294
M.aus	*Melanotaenia australis*	Western rainbowfish	37	3706
M.cyp	*Megalops cyprinoides*	Tarpon	9	44
N.ate	*Neosilurus ater*	Narrow‐fronted catfish	1	5
N.ere	*Nematalosa erebi*	Bony bream	45	5211
N.gra	*Neoarius graeffei*	Lesser salmon catfish	6	72
N.hyr	*Neosilurus hyrtlii*	Hyrtl's tandan	21	220
O.sel	*Oxyeleotris selheimi*	Giant gudgeon	17	74
P.ren	*Porochilus rendahli*	Rendahl's catfish	8	197
S.kre	*Strongylura krefftii*	Freshwater longtom	8	31
T.kim	*Toxotes kimberleyensis*	Kimberley archerfish	19	209

### Connectivity, persistence, and fish occurrence

**FIGURE 3:**
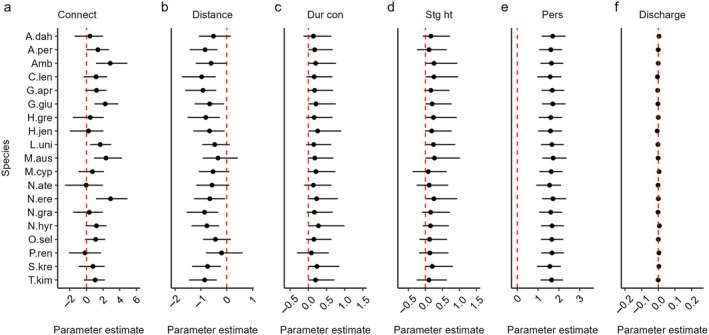
Species‐specific model predicted parameter estimates for (a) connection to river, (b) pool distance to river, (c) duration of connectivity, (d) river stage height, (e) pool persistence during previous dry season, and (f) total main channel discharge two wet seasons prior. The dashed line represents effect size of zero, that is, no effect. Error bars indicate 90% Bayesian credible intervals at the species level. Error bars that do not overlap zero indicate significance at α≤0.1. See Table 1 for species codes.

**FIGURE 4:**
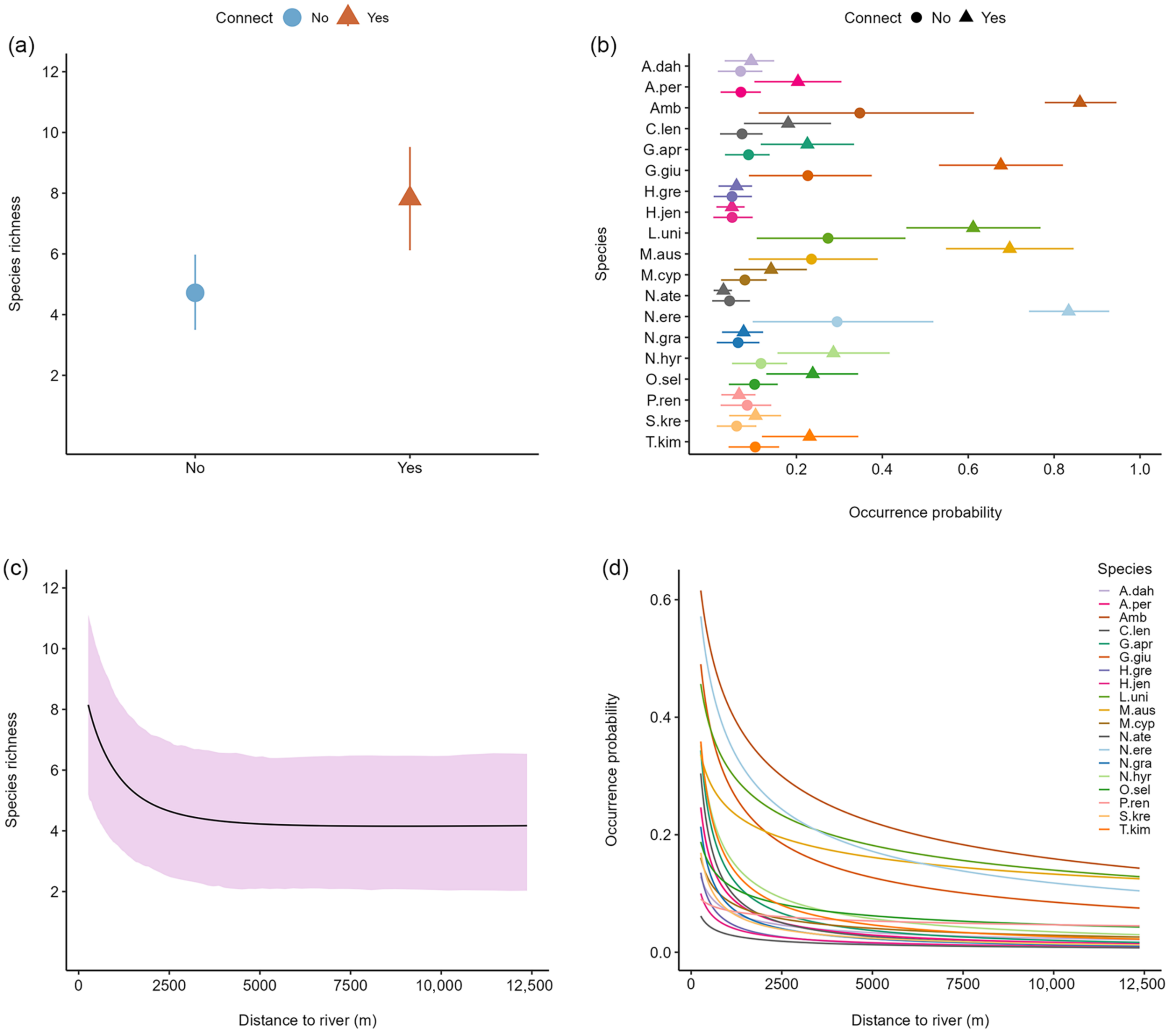
Model predicted community (a, c) and species‐specific (b, d) covariate effects for (top panels) connection to river during the preceding wet season and (bottom panels) pool distance to river. Shaded area and error bars represent 90% Bayesian credible intervals. See Table 1 for species codes.

Posterior probability distributions indicated that lateral connectivity metrics influenced species‐level occurrence, but the strength of relationships varied with species and metric (Table 2). For instance, connection to the river increased occurrence probabilities of all species by 16.8 to 66.4% (mean 38.8%) except for *N. ater*, *Porochilus rendahli*, and *H. jenkinsi*. However, the effect of connection was only statistically significant at α≤0.1 for the four most common species (*Ambassis* spp., *L. unicolor*, *M. australis*, and *N. erebi*) and *Glossogobius giuris* (Figure 3). The positive influence of connection to the river at the species level also manifests at the community level, where mean predicted species richness was 7.83 in pools that connected compared to 4.72 in pools that did not connect (Figure 4a). This indicates that fish species richness near doubled when floodplain pools connected to the river during the wet season.

**TABLE 2:** Results from model selection at the community level.

Variable	Effect size	SD	LCI	UCI	w	*P*(>0)
Connection to river in preceding wet season	1.121	0.557	0.151	1.982	1.000	0.971
Distance to river	−0.650	0.188	−0.955	−0.347	1.000	0.000
Duration of connectivity	0.742	1.373	−0.884	3.863	0.525	0.830
River stage height	0.902	1.789	−1.636	4.886	0.478	0.810
Persistence over preceding dry season	1.642	0.263	1.219	2.073	1.000	1.000
Total main channel discharge two wet seasons prior	−0.973	2.185	−4.467	2.258	0.047	0.386

*Note*: LCI is 90% lower Bayesian credible interval; UCI is 90% upper Bayesian credible interval; w is the inclusion parameter that is, the probability that each covariate is in the best predictive model; and *P*(>0) is the probability of a covariate effect greater than zero. Note, *P*(>0) for covariate “distance to river” is zero indicating a probability of 1 that the effect was negative (see Appendix S1: Figure S2b). Bayesian credible intervals that do not overlap zero indicate significance at α≤0.1.

When pools connected to the river, the occurrence probability of all species was negatively influenced by distance between floodplain pools and the river (Table 2). This effect was particularly strong in pools less than 3000 m from the river but decayed such that it was not noticeable at distances greater than 5000 m (Figure 4d), suggesting that the effect of distance on dispersal and recolonization diminishes with increasing distance from the river. The strength of this relationship was also species‐specific. Occurrence probabilities of *Amniataba percoides*, *Craterocephalus lentiginosus*, *Glossamia aprion*, *G. giuris*, *H. greenwayi*, *H. jenkinsi*, *N. erebi*, *Neoarius graeffei*, *Neosilurus hyrtlii*, *S. krefftii*, and *Toxotes kimberleyensis* in floodplain pools were all significantly greater close to the river (Figure 3b). Occurrence probabilities for common species (*Ambassis* spp., *L. unicolor*, *M. australis*, *N. erebi*) remained higher than those of other species, as distance to the river increased (Figure 4d), suggesting these species are strong dispersers and more likely to occur in pools further from the river. At the community level, a similar pattern manifests with species richness decreasing rapidly with increasing distance to the river up to approximately 2000 m but decaying strongly at distances >3000 m (Figure 4c).

**FIGURE 5:**
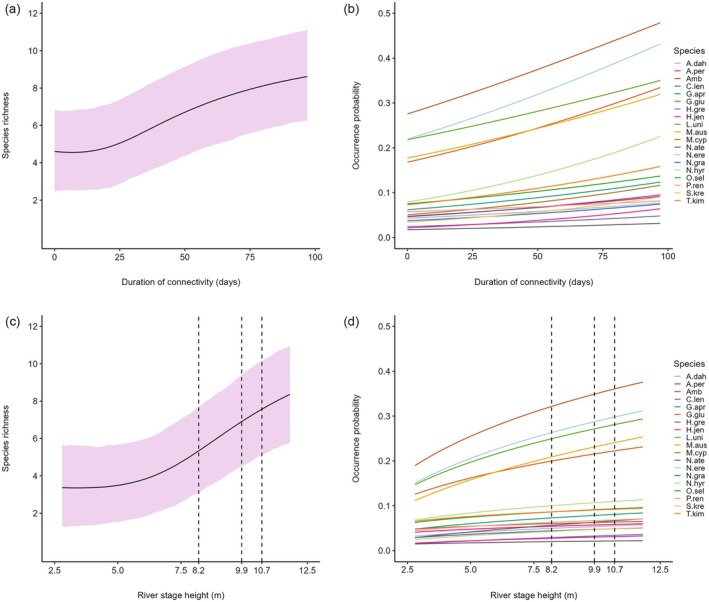
Model predicted community (a, c) and species‐specific (b, d) covariate effects for (top panels) duration of connectivity and (bottom panels) river stage height. Vertical dashed lines in (c) and (d) represent approximate stage heights of minor (8.2 m), moderate (9.9 m), and major (10.7 m) overbank inundation of the floodplain (Bureau of Meteorology, 2024). Shaded areas and error bars represent 90% Bayesian credible intervals. See Table 1 for species codes.

There was also evidence that the duration of connectivity and river stage height influenced fish assemblage structure, but support was not as strong as for other connectivity metrics. Species‐specific mean effects for the duration of connectivity and river stage height were positive (Figure 3c,d, respectively), suggesting that longer periods of connection to the river and high river water levels increased occurrence probabilities of all species. At the community level, high upper credible intervals indicate the effect of both covariates could be substantial (Table 2). However, these effects were not statistically significant at the species level (Figure 3c,d) or community level (Table 2). Nonetheless, patterns were present that are useful for decision makers. For instance, the posterior probability that the duration of connectivity had an effect greater than zero was 0.830 (Table 2), indicating that in 83% of cases, increasing the duration of connectivity had a positive influence on community‐level species occurrence (see Appendix S1: Figure S2c). A notable increase in fish diversity was observed when the connection persisted for more than 25 days with predicted species richness increasing from 5.02 at 25 days to 6.70 at 50 days and 8.40 at 90 days (Figure 5a). The posterior probability for an effect greater than zero for river stage height was 0.810 (Table 2), suggesting >80% chance that increasing river stage height had a positive influence on community‐level occurrence (see Appendix S1: Figure S2d). Indeed, when water levels exceeded ~6 m, species richness in floodplain pools increased notably from 3.78 to 8.36 at a maximum recorded stage height of 11.81 m (Figure 5c).

**FIGURE 6:**
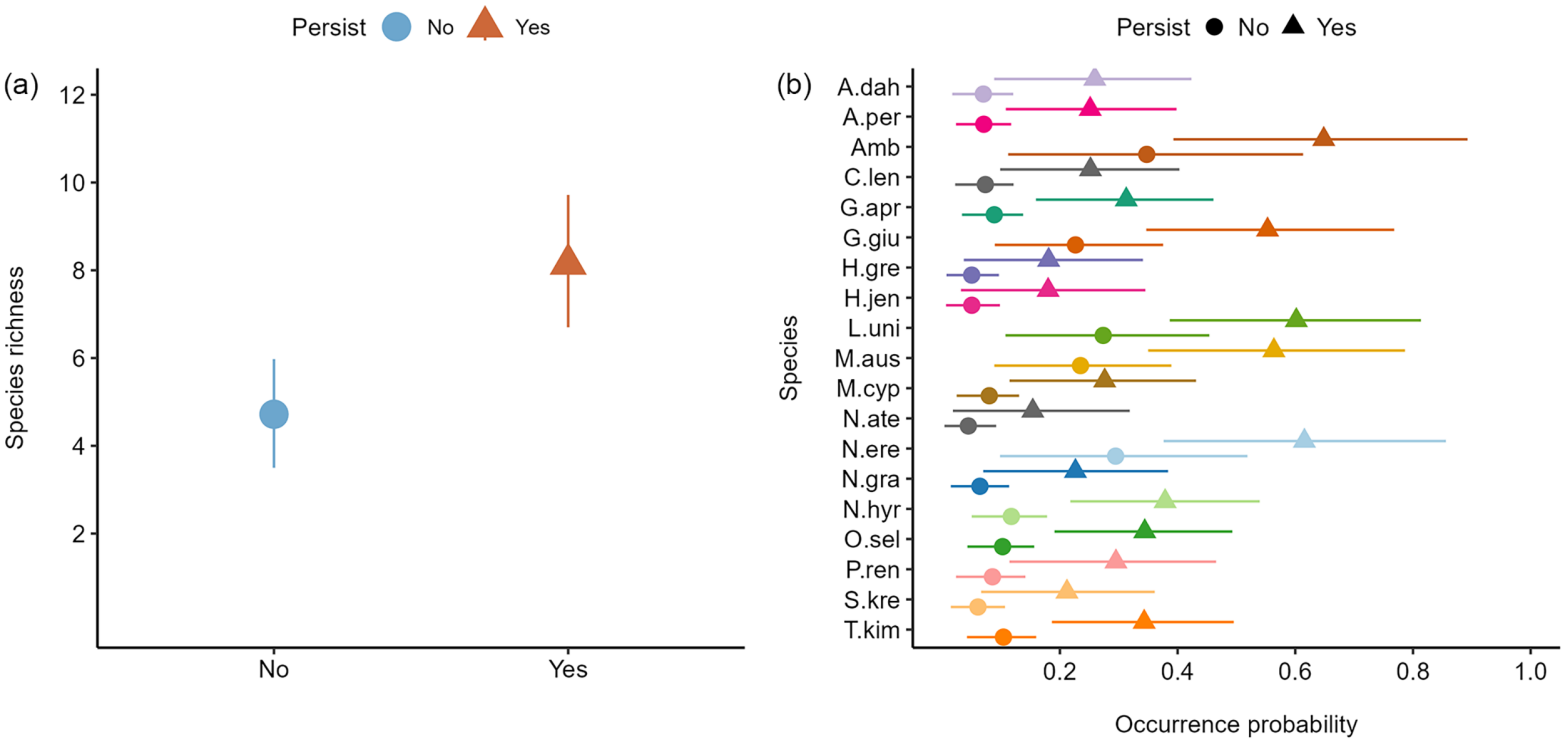
Model‐predicted community (a) and species‐specific (b) covariate effects for pool persistence during the previous dry season. Shaded area and error bars represent 90% Bayesian credible intervals. See Table 1 for species codes.

In addition to lateral connectivity, we found mixed evidence that other factors linked to hydrology influenced fish assemblage structure in floodplain pools, with strong support for pool persistence but no support for total main channel discharge two wet seasons prior. Pool persistence over the preceding dry season was the only covariate assessed that had a strong positive effect on the occurrence of all species (Figure 3e). Pools that persisted were predicted to support 8.14 species compared to 4.72 in pools that dried (Figure 6a), with individual species occurrence probability likely to be between 86% and 271% higher, depending on the species (Figure 6b). This result highlights the importance of dry season extirpation on floodplain fish assemblages. Total main channel discharge two wet seasons prior had a negligible effect on species and community‐level occurrence as indicated by species‐specific mean effect sizes close to zero (Figure 3f), Bayesian credible intervals that overlapped zero, and a low inclusion probability in the best predictive model (Table 2).

### Sampling and fish detection

Gear type influenced detection probability at both the community and species levels. The 10 m seine net was the most effective method of describing fish assemblages with a mean detection probability of 0.658 (Figure 2d). The 7 m seine net which had the smallest mesh size (2 mm) also had a high mean detection probability at the community level (0.599) and was particularly effective at detecting the presence of *Ambassis* spp., *M. australis*, and *C. lentiginosus* (Figure 2c). Boat electrofishing was the least efficient method of sampling, with a mean detection probability of 0.223 across all species (Figure 2e).

## DISCUSSION

This study quantifies the relationship between wet season and dry season components of the hydrological cycle and floodplain fish assemblages to inform the management of a river threatened by water resource development and climate change. Our results demonstrate that lateral connectivity during the wet season influenced the occurrence of fish in floodplain pools. Pools that were connected to the river by short distances were substantially more species rich than distal pools, an effect that was particularly strong at distances <2000 m. The duration of connectivity and river stage height were less influential, but generally pools connected to the river for longer periods contained more species, as did those exposed to higher river stage height during the preceding wet season. A threshold effect was observed for these two connectivity metrics with connection greater than 25 days and stage height >6 m needed before species richness increased. We found strong evidence for a dry season influence on fish assemblage structure in floodplain pools. Pool persistence during the preceding dry season had the largest effect on fish species richness and was the only covariate to significantly affect the occupancy probability of all species. We found no evidence for an effect of antecedent flow conditions, for example, total main channel discharge two wet seasons prior. Our findings provide insight into important ecological processes structuring fish in floodplain pools which can be used to assist the development of water planning policy.

During the wet season, dispersal typically plays a key role in structuring fish assemblages in tropical floodplain habitats (Fernandes et al., 2009, 2014). This is consistent with findings in the Fitzroy River, where wet season connectivity metrics strongly influenced fish assemblage structure. For example, the distance between floodplain pools and the river had a strong negative influence on occurrence and species richness. Given the brevity of river‐floodplain connectivity observed in the Fitzroy River (days–weeks) (Jardine, Pettit, et al., 2012), it is probable that many fishes were unable to access distal pools before disconnection occurred. When lateral connectivity did persist for long periods, specifically for more than 25 days, there was an increase in species richness across the floodplain and species‐specific occurrence probabilities increased. Both species richness and occurrence probability continued to increase to a maximum recorded duration of connectivity of 90 days, indicating extended periods of connection facilitate exchange between habitat patches. We also observed a hydrological component to connectivity. Species richness in floodplain pools increased notably when river stage height surpassed approximately 6 m and continued to increase to a maximum recorded stage height of 11.81 m. In the river's lower reaches, minor overbank inundation of the floodplain during the wet season occurs on average at water levels of approximately 8.2 m with moderate and major flooding at 9.9 and 10.7 m, respectively (Bureau of Meteorology, 2024). River stage height of ~6 m likely represents the average height at which floodplain distributary creeks start to flow. During within‐bank flood events, fish movement is restricted to connected back‐channels and flood‐runner creeks. These restrictions will be removed during overbank flood events, allowing fish to navigate freely across the inundated floodplain. Our results suggest that high river stage height resulting in large overbank flood events is important for maximizing fish species richness across the floodplain. These findings are consistent with existing literature in both tropical and temperate river systems describing the influence of dispersal limitation on fish community structure (Arrington et al., 2005; Fernandes et al., 2014; Miranda, 2005).

Surface water connectivity during the wet season is important for shaping fish communities; however, different components of the hydrological cycle can also substantially influence community dynamics (Kennard et al., 2007; Ruetz et al., 2005). In this study we demonstrate that periods of disconnection and drying play an important role in shaping fish assemblages in floodplain pools of the Fitzroy River. Pools that persisted the entire duration of the dry season prior to wet season river‐floodplain connection supported fish assemblages with twice the species richness of pools that dried. Indeed, whether a pool dried or persisted had the strongest effect on species richness out of all model covariates. Pool persistence has been demonstrated to be a strong predictor of species richness and fish assemblage structure in other intermittent river systems in northern Australia (Beesley & Prince, 2010) and elsewhere (Baber et al., 2002; Ruetz et al., 2005). Moreover, floodplain pools that persist during the dry season may also provide important refuges which can act as “stepping‐stones” to more distal habitat patches when connection is reestablished (Holt, 1993; Magoulick & Kobza, 2003). The persistence of semi‐permanent riverine pools is often strongly influenced by antecedent flow conditions (Leigh et al., 2010) which can also affect fish assemblages due to the way in which it structures the spatial distribution of species across the landscape and its influence on recruitment dynamics (Balcombe & Arthington, 2009; Fornaroli et al., 2020; Humphries et al., 1999; Whiterod et al., 2015). We found no evidence for the direct influence of total main channel discharge two wet seasons prior in shaping fish communities on the floodplain of the Fitzroy River. However, in our model, this covariate was structured as an interactive effect that was only relevant if pools persisted throughout the dry season. Our results suggest that in the Fitzroy River, antecedent flow conditions primarily shape fish assemblages via whether pools persist or dry before river‐floodplain connectivity is re‐established.

Habitat‐related niche processes and species functional traits may also interact with hydrological processes that influence dispersal and extirpation to structure floodplain fish assemblages (Arrington et al., 2005; Hoeinghaus et al., 2003). A strong negative relationship with distance to the river was observed for species commonly associated with main channel habitats, for example, *A. percoides*, *G. aprion*, *H. greenwayi*, *H. jenkinsi*, *N. graeffei*, *S. krefftii*, and *T. kimberleyensis* (Gwinn et al., 2024; Lear et al., 2023), suggesting species‐specific habitat associations may have also influenced fish assemblage structure in the Fitzroy River. Several species in northern Australia are known to use floodplain habitats for foraging or as nurseries but return to deep water refuges during the wet to dry transition (Crook et al., 2020; Jardine, Pusey, et al., 2012). This may reflect an absence of suitable dry season refugia in distal floodplain pools for some species, providing indirect evidence that habitat‐related niche processes may interact with dispersal processes, which have previously been observed in the Fitzroy River (Warfe et al., 2013). Common species (*Ambassis* spp., *L. unicolor*, *M. australis*, *N. erebi*) were not strongly influenced by distance to the river, suggesting these species are strong dispersers and habitat generalists, a finding consistent with other systems in northern Australia (Pusey et al., 2017). The presence of these species in distal floodplain pools also suggests environmental filtering may play a role in structuring floodplain fish assemblages. For instance, during the dry season shallow pools that contract under drying conditions often suffer deteriorating water quality and habitat loss which can favor species with environmentally resistant traits (Arthington et al., 2005; Sargent & Galat, 2002). *Ambassis* spp., *L. unicolor*, *N. erebi*, and other *Melanotaeniidae* are routinely found surviving and breeding in the most marginal habitats (Kerezsy et al., 2013; Pusey et al., 2004), indicating adaptations related to environmental resilience may buffer against the effects of harsh environmental conditions, favoring proliferation of certain species or life‐history strategies across the floodplain (Chen et al., 2022; Thomaz, 2022; Winemiller, 1989).

Biotic interactions can further shape fish assemblages in drying floodplain pools, compounding the effects of abiotic stressors. Increased fish density in shrinking pools can intensify competition for resources, increase predator–prey interactions, and increase disease transmission (Magoulick & Kobza, 2003). Shallow water also exposes fish to greater risk from avian predators (Gawlik, 2002). Although we did not include such processes in our model to avoid over‐parameterization, future studies should investigate these biological factors to better understand their role in shaping community dynamics in the Fitzroy River.

When sampling fish communities, detection is imperfect and may be influenced by a variety of factors (Gwinn, Beesley, et al., 2016). For instance, the type of sampling gear used is often biased, preferentially targeting particular species and life history strategies (Beesley et al., 2014; Wedderburn, 2018). Our results are consistent with this literature as indicated by species‐specific detection probabilities that vary with gear type. On average, the 10 m seine net was the most efficient gear for sampling fish communities in floodplain pools on the Fitzroy River. The 7 m seine net, with a smaller mesh size, outperformed all other gear when sampling small‐bodied species such as *Ambassis* spp., *M. australis* and *C. lentiginosus*. In general, all gear types were most effective at sampling common species, suggesting that detection may have been related to underlying patterns of species abundance (McCarthy et al., 2013). Our results highlight that accounting for imperfect detection is vital for deriving unbiased estimates of fish communities on the floodplain of the Fitzroy River.

In the face of water resource development in a changing climate, understanding how wet season and dry season components of the hydrological cycle influence fish assemblages on the floodplain is crucial for maintaining healthy ecosystem functioning (Stoffels et al., 2022). Quantifying these relationships can facilitate the assessment of management decisions aimed at protecting or restoring fish diversity. In this study we provide quantitative estimates of wet season lateral connectivity–fish relationships and highlight the importance of dry season pool persistence in structuring fish assemblages on the floodplain of the Fitzroy River. Our findings suggest that fish species richness can be increased, particularly in pools within 2000 m from the river (along flow pathways), when wet season flows are sufficient to connect the river to the floodplain for more than 25 days and when pools persist throughout the preceding dry season. Indeed, pools that persist are not only more species rich the following wet season, but they may also provide important dry season refugia that facilitate colonization to more distal floodplain habitats (Magoulick & Kobza, 2003; Thomaz, 2022). This finding is particularly relevant in a region where conditions that exacerbate pool drying are expected to intensify (Suppiah et al., 2007). When pools do persist, species richness is maximized by expansive and protracted flooding, for example, when river stage height exceeds 11 m and when river–floodplain connectivity lasts 90 days. However, given that large and protracted flood events are less likely to be impacted by water take, we recommend that managers seeking to maintain floodplain fish diversity ensure that water resource development does not unduly increase the likelihood that floodplain pools dry.

## AUTHOR CONTRIBUTIONS

*Conceptualization and developing methods*: Oliver P. Pratt, Leah S. Beesley, Daniel C. Gwinn, Michael M. Douglas. *Conducting the research*: Oliver P. Pratt, Leah S. Beesley, Chris S. Keogh, Bradley J. Pusey. *Data analysis and data interpretation*: Oliver P. Pratt, Daniel C. Gwinn, Leah S. Beesley, Thiaggo C. Tayer. *Preparation of figures and tables*: Oliver P. Pratt, Daniel C. Gwinn, Leah S. Beesley. *Writing*: Oliver P. Pratt, Leah S. Beesley, Bradley J. Pusey, Daniel C. Gwinn, Samantha A. Setterfield, Michael M. Douglas.

## CONFLICT OF INTEREST STATEMENT

The authors declare no conflicts of interest. Recommendations regarding water planning policy are the opinions of the authors and not the State Government of Western Australia.

## Supporting information


Appendix S1.

## Data Availability

Data (Pratt, 2025) are available in the University of Western Australia's research repository at https://doi.org/10.26182/hn8y-pb39.
