# Supplementary material for: Large, prolonged flooding and pool persistence promote floodplain fish diversity in a threatened river
Source: Ecol Appl. 2025 Nov 25;35(7):e70155. doi: 10.1002/eap.70155 (PMC12646871; doi:10.1002/eap.70155)
Supplement: Supplementary file 1 — Appendix S1. [file EAP-35-e70155-s001.pdf]

Large, prolonged flooding and pool persistence promote floodplain fish diversity in a threatened river

Oliver P. Pratt, Leah S. Beesley, Daniel C. Gwinn, Thiaggo C. Tayer, Bradley J. Pusey, Chris S. Keogh, Samantha A. Setterfield and Michael M. Douglas.

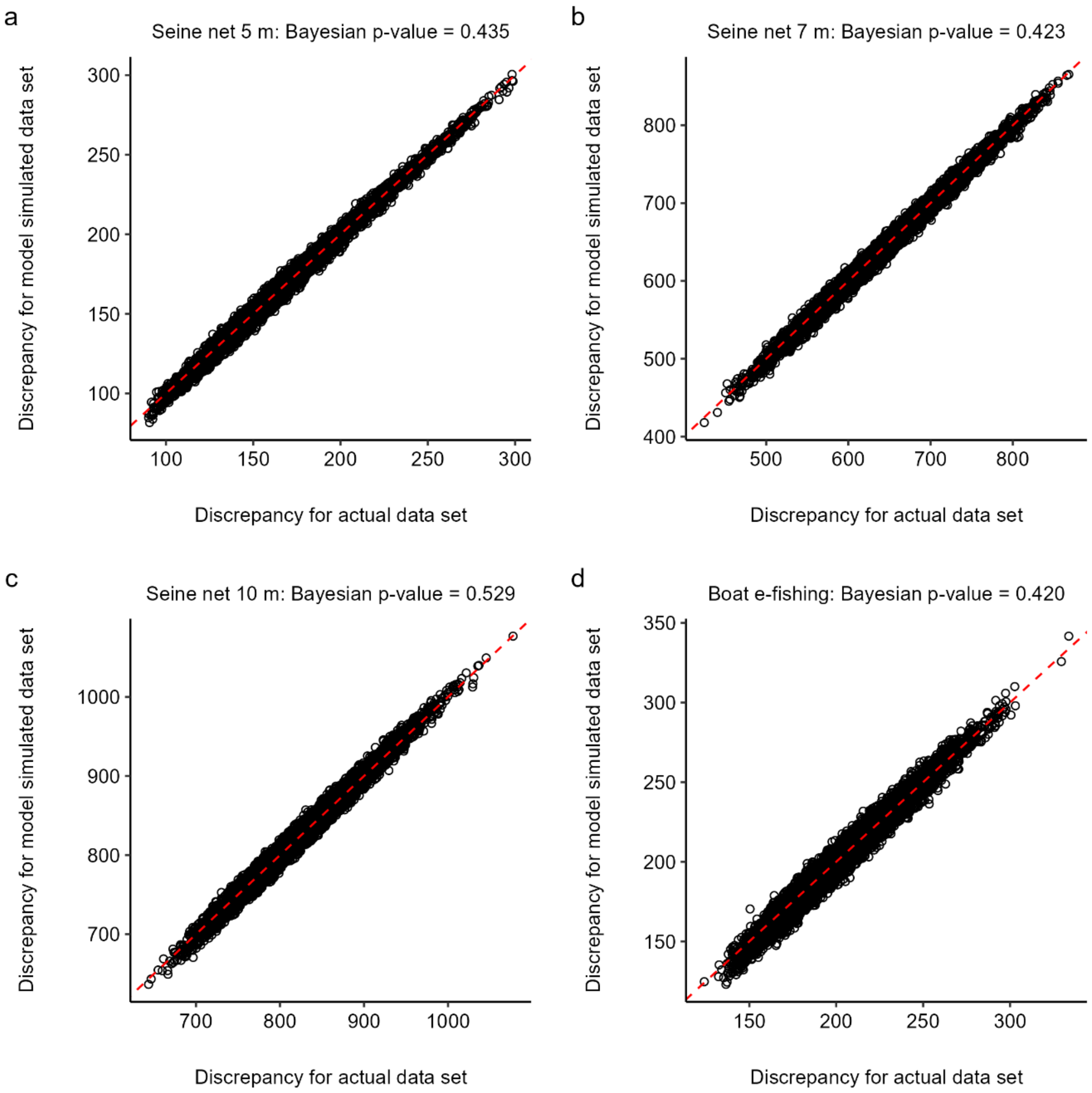

Figure S1- Posterior check of detection model fit using Bayesian p-values for (a) 5 m seine net, (b) 7 m seine net, (c) 10 m seine net, and (d) boat e-fishing. Even spread of fit statistic derived from model simulated data and actual data around the 1:1 line (dashed line) indicates adequate model fit.

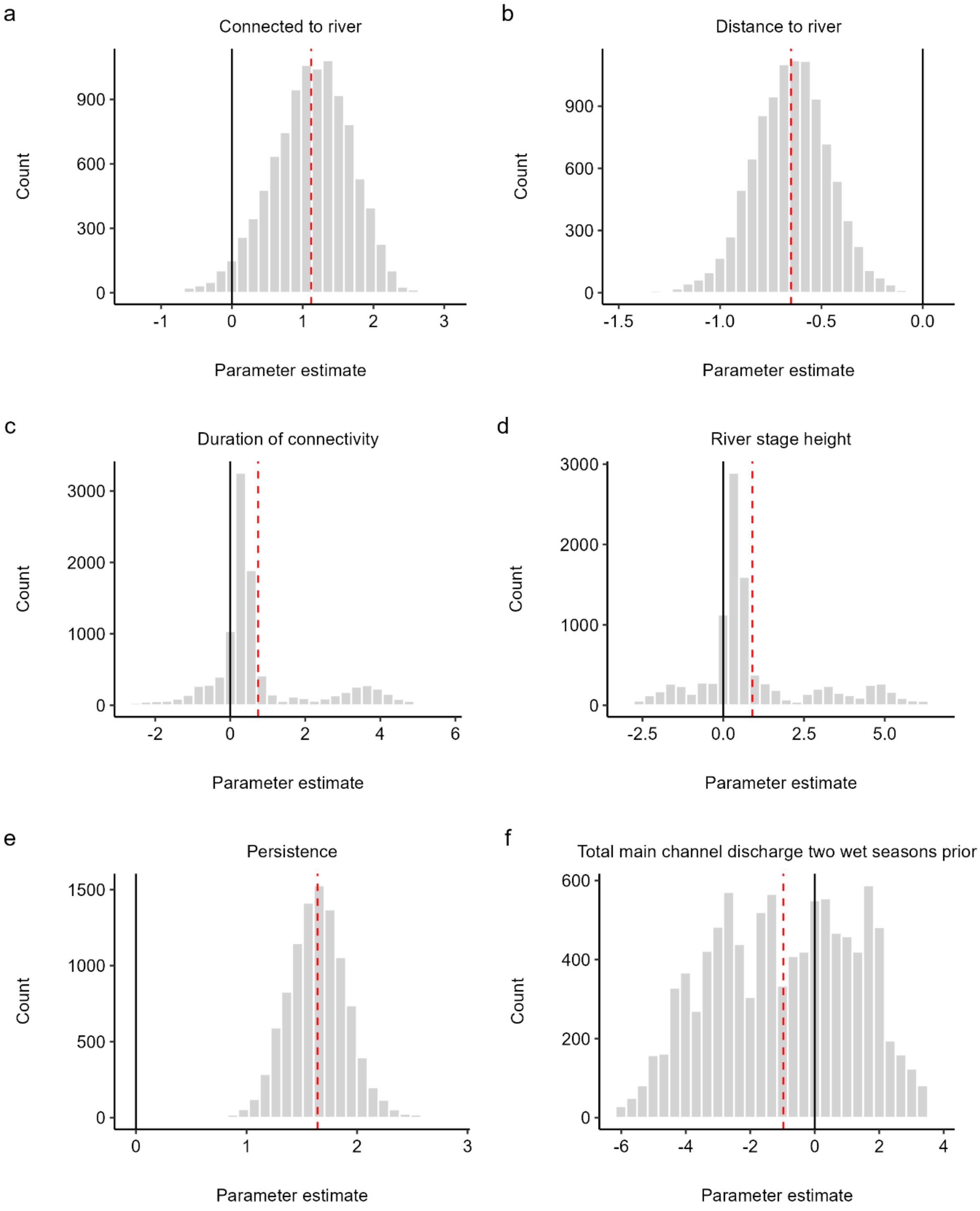

Figure S2- Histograms of posterior sample (10 000 iterations) of parameter estimates for (a) connection to river in preceding wet season, (b) pool distance to river, (c) duration of connectivity, (d) river stage height, (e) pool persistence during previous dry season, and (f) total main channel discharge two wet seasons prior. Solid lines represent zero effect and dashed line the mean/ community-level parameter estimate.
